# Brain structural connectivity and neuroticism in healthy adults

**DOI:** 10.1038/s41598-018-34846-1

**Published:** 2018-11-07

**Authors:** Issei Ueda, Shingo Kakeda, Keita Watanabe, Koichiro Sugimoto, Natsuki Igata, Junji Moriya, Kazuhiro Takemoto, Asuka Katsuki, Reiji Yoshimura, Osamu Abe, Yukunori Korogi

**Affiliations:** 1 Department of Radiology, University of Occupational and Environmental Health, Fukuoka, Japan; 2 Department of Bioscience and Bioinformatics, Kyushu Institute of Technology, Fukuoka, Japan; 3 Department of Psychiatry, University of Occupational and Environmental Health, Fukuoka, Japan; 4 Department of Radiology, Graduate School of Medicine, University of Tokyo, Tokyo, Japan

## Abstract

Understanding the neural correlates of the neurotic brain is important because neuroticism is a risk factor for the development of psychopathology. We examined the correlation between brain structural networks and neuroticism based on NEO Five-Factor Inventory (NEO-FFI) scores. Fifty-one healthy participants (female, n = 18; male, n = 33; mean age, 38.5 ± 11.7 years) underwent the NEO-FFI test and magnetic resonance imaging (MRI), including diffusion tensor imaging and 3D T1WI. Using MRI data, for each participant, we constructed whole-brain interregional connectivity matrices by deterministic tractography and calculated the graph theoretical network measures, including the characteristic path length, global clustering coefficient, small-worldness, and betweenness centrality (BET) in 83 brain regions from the Desikan-Killiany atlas with subcortical segmentation using FreeSurfer. In relation to the BET, neuroticism score had a negative correlation in the left isthmus cingulate cortex, left superior parietal, left superior temporal, right caudal middle frontal, and right entorhinal cortices, and a positive correlation in the bilateral frontal pole, left caudal anterior cingulate cortex, and left fusiform gyrus. No other measurements showed significant correlations. Our results imply that the brain regions related to neuroticism exist in various regions, and that the neuroticism trait is likely formed as a result of interactions among these regions. This work was supported by a Grant-in-Aid for Scientific Research on Innovative Areas (Comprehensive Brain Science Network) from the Ministry of Education, Science, Sports and Culture of Japan.

## Introduction

The Five Factor Model (FFM) is one of the most widely accepted taxonomies of personality and includes various aspects of social behavior and emotional responsiveness: openness, conscientiousness, extraversion, agreeableness, and neuroticism. Among these, neuroticism has been widely recognized in various theoretical approaches to human personality^1^. Neuroticism is characterized by a tendency to worry and be anxious^2^ and is related to the experience of having a negative affect^1,3,4^. Many previous studies have demonstrated that neuroticism is associated with depressive symptoms or depression. A large meta-analysis reported higher levels of neuroticism in individuals suffering from depression than in healthy controls^5^. In another meta-analysis, a longitudinal association was observed between high neuroticism and depressive symptoms or depression^6^. Because neuroticism is a potential risk factor for the onset of psychopathology, recent research has focused on understanding the neural correlates of the neurotic brain^7–10^.

Knowledge of the relationships between white matter (WM) integrity and FFM personality traits will help us understand how the integrity of the anatomical connections in the brain relates to emotion, cognition, and behavior^11,12^. Diffusion tensor imaging (DTI) is a useful magnetic resonance imaging (MRI) technique for quantifying and describing the microstructural changes in the WM. Previous DTI studies demonstrated that neuroticism is correlated with fractional anisotropy (FA) and mean diffusivity (MD) in the anterior cingulum or uncinate fasciculus^8^. Notably, a study with a large sample size (668 participants) showed that higher levels of neuroticism were significantly associated with lower FA in the uncinate, suggesting that higher neuroticism is associated with reduced structural connectivity between the prefrontal cortex and the amygdala^13^. Previous studies by functional MRI (fMRI) also demonstrated alterations in the frontal-limbic circuitry in association with neuroticism^10^. On the other hand, previous studies showed an association between decreased WM integrity and neuroticism not only in the fiber tracts interconnecting the prefrontal cortex and amygdala, but also in multiple other fiber tracts^10,14^. The resting-state functional MRI (rs-fMRI) study by Servaas *et al*. also showed that the cingulo-operculum subnetwork demonstrated more ties with other functional subnetworks in association with neuroticism, whereas cognitive control networks in the default mode network showed less efficient information processing^14^. Thus, the previous evidences suggest that the brain regions related to neuroticism exist over various regions.

Imaging connectomics, which can evaluate interregional structural and functional connectivity patterns, has opened new avenues towards understanding the organization and function of the human brain^15,16^. The brain is believed to support global and local information communication through an integrative network^17^. Using a graph theory analysis, recent studies on connectomics have demonstrated a number of non-trivial topologic features in whole-brain networks, including efficient small world architecture, a prominent modular structure, and highly connected and centralized network hubs^18,19^. We therefore used whole-brain network models to gain insight into this potentially high-dimensional interplay among the brain regions behind the neuroticism trait. In this study, using DTI data from healthy adults, we examined the tract-based whole-brain network measures to determine whether a correlation exists between structural network organization and neuroticism score on the NEO Five-Factor Inventory (NEO- FFI), which is a widely used measure of FFM.

## Methods

### Study Participants

We acquired both MRI and self-reported NEO-FFI data from 51 healthy volunteers (male, n = 33; female, n = 18; mean age, 38.5 years; range, 20–65 years; standard deviation, 11.7 years) without any history of significant head injury, seizure, or neurologic condition. The subjects were eligible to participate in the protocol if they have never been diagnosed with an axis I or II psychiatric disorder, as confirmed by the Structured Clinical Interview for the DSM-IV (SCID)^20^, and if they had no history of psychotropic medication use within the preceding six months. This study was approved by the ethics committee of the University of Occupational Environmental Health. All participants gave their written informed consent to participate in the study.

### Five Factor Model Personality Traits

The self-reported version of the revised NEO- FFI (Japanese version) was used to assess personality traits^21^. The NEO- FFI generates one score each for openness, conscientiousness, extraversion, agreeableness, and neuroticism. In this study, we only used the neuroticism data. The R software program (version 3.3.1; www.R-project.org) was used to calculate Cronbach’s alpha values for the neuroticism trait.

### Magnetic Resonance Imaging

MRI data were obtained using a 3 T scanner (Signa EXCITE 3 T; GE Healthcare, Milwaukee, WI, USA) with a dedicated eight-channel, phased-array coil (USA Instruments Aurora, OH, USA). All participants underwent a brain MR examination, which included three-dimensional (3D) T1-weighted imaging (T1Wl) and DTI. 3D T1Wl was obtained by 3D fast-spoiled gradient-recalled (3D FSPGR) acquisition at a steady state. The parameters for the 3D FSPGR were as follows repetition, 10 ms; echo, 4.1 ms; inversion, 700 ms; flip angle, 10°; field of view, 24 cm; section thickness, 1.2 mm; and resolution, 0.9 × 0.9 × 1.2 mm. The DTI was acquired by a single-shot, spin-echo planar sequence with the following parameters: TR/TE, 12000/83.3 ms; slice thickness, 4 mm; no gap; field of view, 26 cm; number of excitations, 1; and spatial resolution, 1.02 × 1.02 × 4 mm. Diffusion gradients (b value of 1,000 s/mm^2^) were simultaneously applied for each of the three axes around a 180-degree pulse. The diffusion properties were measured in 25 non-collinear directions.

### Image Processing (Network Construction and Calculation of Graph Theory Metrics)

**Figure 1:**
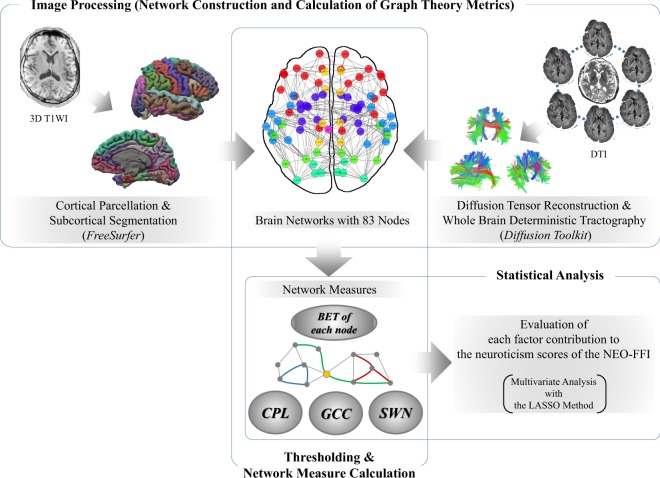
Overview of the data processing and analysis. 3D T1WI, three-dimensional T1-weighted imaging; DTI, diffusion tensor imaging; CPL, characteristic path length; GCC, global clustering coefficient; SMW, small-worldness; BET, betweenness centrality; NEO-FFI, NEO Five-Factor Inventory; LASSO, least absolute shrinkage and selection operator.

We processed the 3D T1Wl and DTI data from each participant using the Connectome Mapper pipeline software program^22,23^ (Fig. 1). First, we used the affine registration in the eddy correct tool that was implemented in the software program from the Oxford Centre for fMRI of the Brain (FSL, FMRIB Software Library, http://www.fmrib.ox.ac.uk/fsl/) to correct each diffusion-weighted image for distortion caused by head motion and eddy currents.

We used the FreeSurfer software program (Version 5.3; http://surfer.nmr.mgh.harvard.edu) to parcellate the cortical surface, segment the gray matter and WM, and define 83 regions of interest, which included 41 regions in each hemisphere and 1 region in the brainstem, with the Desikan–Killiany Atlas^24^. The regions were transformed into the DTI space using boundary-based linear registration. All processed images were inspected for any artifacts, segmentation, or registration errors. Diffusion tensor reconstruction and whole brain deterministic tractography were performed with the Diffusion Toolkit software program (http://www.trackvis.org/dtk) based on the fiber assignment by the continuous tracking algorithm, which had a threshold angle of 60°, an auto mask threshold, with the application of the spline filter, and no additional options.

Finally, the adjacency matrix ***A*** with 83 × 83 entries (i.e., the weighted network with 83 nodes) was generated for each subject, with *A*_*ij*_ corresponding to the weighted connectivity between structures *i* and *j*.

### Thresholding

According to a previous study^25^, the matrices or weighted networks were thresholded and binarized in order to emphasize the differences between strong and weak interactions (i.e., to minimize the noise in brain connectivity) (Fig. 1). The procedures were also performed to obtain networks with the same number of edges among the subjects, because the majority of the network measures were highly sensitive to the number of edges^26^; in particular, the threshold *K* indicated that the strongest *K* edges were included in each network. Threshold *K* was selected to maximize the difference between the actual and randomized networks in the context of information theory. For all possible node pairs, we calculated the probability that edges would occur in the actual and randomized networks. We compared the Shannon entropy, which was calculated based on the probabilities, between the actual and randomized networks to determine the optimum *K* at which the maximum difference in entropy occurred^25^. The randomized networks were generated from an actual network using a degree-preserving method^27^. At each threshold, we repeatedly generated one randomized network from a randomly selected actual network to create 500 random networks. In this study, the optimum *K* was 343 (i.e., the average node degree was ~8.3).

### Network Measure Calculation

We calculated the network measures of the 51 binarized brain networks (i.e., interregional connectivity) using the R software program (version 3.3.1; www.R-project.org) and R-package *igraph* (version 1.0.1; igraph.org). Based on previous studies^28^, we obtained the characteristic path length (CPL), global clustering coefficient (GCC), small-worldness (SMW), and betweenness centrality (BET) of each node (Fig. 1). CPL was defined as the shortest average path length among all reachable node pairs and was calculated using the *average.path.length* function in the *igraph* package. GCC was defined as the average nodal clustering coefficient, which was defined as the ratio of the number of edges among the neighbors of a node to the total number of possible connections among the neighbors. GCC were obtained using the *transitivity* function in the igraph package. The SMW^29^ was proposed as a measure of the small-world property in real-world networked systems^30^. The small-world property means that all node pairs in a network are reachable in a short distance (i.e., the distance expected from random networks), although the network was divided into highly interconnected clusters (i.e., the network was far from random networks). Specifically, the SMW was calculated based on the CPL and GCC in the actual and randomized networks, as follows:

$$({{\rm{GCC}}}_{{\rm{act}}}/{{\rm{GCC}}}_{{\rm{rand}}})/({{\rm{CPL}}}_{{\rm{act}}}/{{\rm{CPL}}}_{{\rm{rand}}})$$

where X_act_ represents a network measure X (i.e., GCC or CPL) in the actual networks. X_rand_ was the average X obtained from 500 randomized networks. The BET of each node was calculated using the *betweenness* function in the *igraph* package^28,31^.

### Statistical analysis

To evaluate the contribution of each factor (i.e., age, sex, CPL, GCC, SWN, and BET of each region) to the neuroticism score of the NEO-FFI, we conducted a multivariate analysis. In this study, we could not use a direct regression model for all 88 explanatory variables (i.e., age, sex, 3 global network measures, and the BET values of the 83 nodes) because of the combinational explosion in the model selection and the multicollinearity that mainly arises from feature overlap among network measures. Thus, following a previous study^32^, to avoid this problem as much as possible, we considered the parameter selection using the least absolute shrinkage and selection operator (LASSO) method, which is thought to be useful for regularization, to increase the interpretability of the regression model for finding significant variables^33^.

The multivariate analysis with the LASSO method was performed using the R software program (version 3.4.1; www.R-project.org). Using the *cv.glmnet* and *glmnet* functions from the *glmnet* R package (www.rdocumentation.org/packages/glmnet), we selected parameters and defined the best subset of network measures to include in a regression model. We then conducted a regression analysis using the *lm* function to evaluate the contribution of age, sex, and the selected network measures to the neuroticism score. We considered the associations between the network measure and neuroticism score to be significant when the associated p-value was <0.01. We removed subject data with a Cook’s distance of more than 3 times the mean from the regression analysis.

## Results

### Neuroticism Score of the NEO-FFI

The mean of neuroticism score on the NEO- FFI was 23.5 (standard deviation: 7.5). Cronbach’s alpha coefficient of the neuroticism score was 0.81, indicating the high internal consistency of this score.

### Network Measures

We examined the contribution of age, sex, CPL, GCC, SWN, and BET of each region to the neuroticism score of NEO-FFI. One participant’s data point was removed from the analysis, because its Cook’s distance was more than 3 times the mean. A multivariate regression analysis (Adjusted R-squared = 0.869, F-statistic p-value < 0.0001) was performed after outlier removal and the parameter selection using the least absolute shrinkage and selection operator (LASSO) method.

**Figure 2:**
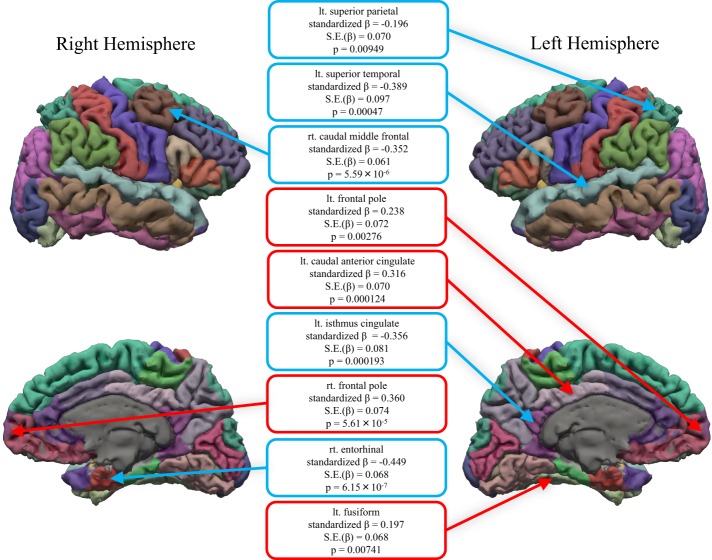
The brain regions for which a significant association was observed between betweenness centrality (BET) and the neuroticism score. The regions with positive and negative associations are indicated with red and blue circles, respectively. lt., left; rt., right; S.E., standard error.

The neuroticism scores were negatively associated with the BET in the left isthmus cingulate cortex, left superior parietal, left superior temporal, right caudal middle frontal and right entorhinal cortices (p < 0.01) (Fig. 2) (Table 1). On the other hand, the neuroticism score was positively associated with the BET in the right frontal pole, left caudal anterior cingulate cortex, left frontal pole, and left fusiform gyrus (p < 0.01) (Fig. 2) (Table 1).

**Table 1:** The association between the neuroticism score and the variables selected by the least absolute shrinkage and selection operator (LASSO) method.

Coefficients	Standardized β	S.E. (β)	t value	Pr (>|t|)
Age	−0.125	0.080	−1.55	0.134
Sex	−0.267	0.161	−1.66	0.109
**BET in brain Regions**				
lt. caudal anterior cingulate	0.316	0.070	4.54	0.000124*
lt. entorhinal	−0.087	0.063	−1.38	0.180
lt. frontal pole	0.238	0.072	3.32	0.00276*
lt. fusiform	0.197	0.068	2.91	0.00741*
lt. isthmus cingulate	−0.356	0.081	−4.37	0.000193*
lt. superior parietal	−0.196	0.070	−2.81	0.00949*
lt. superior temporal	−0.389	0.097	−4.02	0.00047*
rt. caudal anterior cingulate	−0.131	0.073	−1.79	0.0860
rt. caudal middle frontal	−0.352	0.061	−5.74	<0.0001*
rt. cuneus	−0.081	0.060	−1.37	0.184
rt. entorhinal	−0.449	0.068	−6.62	<0.0001*
rt. frontal pole	0.360	0.074	4.82	<0.0001*
rt. lateral orbitofrontal	−0.131	0.071	−1.84	0.0776
rt. lingual	0.080	0.070	1.15	0.261
rt. peri-calcarine	0.141	0.090	1.56	0.130
rt. posterior cingulate	0.053	0.068	0.79	0.439
rt. precuneus	0.019	0.070	0.28	0.783
rt. superior temporal	−0.105	0.105	−1.00	0.329
rt. supramarginal	−0.123	0.064	−1.92	0.0659
lt. caudate	−0.116	0.069	−1.67	0.108
lt. pallidum	−0.107	0.082	−1.31	0.203
rt. caudate	−0.114	0.076	−1.49	0.148

BET, betweenness centrality; LASSO, least absolute shrinkage and selection operator; lt., left; rt., right; S.E., standard error. *Indicates effects that were statistically significant (p < 0.01) at this study assessments.

The multivariate regression analysis showed a residual standard error of 0.363 with 25 degrees of freedom; Multiple R-squared, 0.933; Adjusted R-squared, 0.869; F-statistic, 14.49 with 24 and 25 DF; p-value < 0.0001.

The CPL, GCC, and SMW values were not significantly correlated with the neuroticism score.

## Discussion

The aim of the current study was to investigate the association between the alterations in the structural network organization of the neurotic brain. The BET, which is defined as the fraction of all shortest paths in the network that passes through a node^28^, describes the central nodes that participate in many short paths within a network. Thus, the BET consequently acts as an important control of the information flow^34^, and is useful for detecting important anatomical or functional connections^28^. Our study showed that in comparison to individuals with low neuroticism, highly neurotic individuals had lower BET values in the left isthmus cingulate cortex, left superior parietal, left superior temporal, right caudal middle frontal and right entorhinal cortices, whereas information processing in the neurotic brain predominantly occurred in the right frontal pole, left caudal anterior cingulate cortex, left frontal pole, and left fusiform gyrus. Our results showed regional laterality (more on left side), which is consistent with results from the previous DTI studies; the fiber tracts associated with neuroticism showed asymmetry (more on left side)^8,10^. Although the reason is unclear, it is suggested that there may be laterality in the brain regions affected by the neuroticism trait.

Regarding the brain regions in which a negative association was observed between the neuroticism score and the BET, our results supported the findings of many previous DTI studies^8,10,13,35,36^. Many studies have shown a negative association between the neuroticism score and the FA in the uncinate fasciculus and cingulate cortex^8,13^. The uncinate fasciculus is an association tract that interconnects the inferior frontal gyrus and parts of the limbic system, such as the hippocampus and amygdala. The entorhinal cortex, which was detected in this study, is a part of the anterior parahippocampal gyrus^37^ and an important pathway connecting the amygdala and the hippocampus^38^. The entorhinal cortex is also involved in nociceptive processing and the generation of pain perception^39^, and plays an important role in anxiety^40,41^. Ploghaus *et al*.^39^ found that anxiety-induced hyperalgesia is associated with the activation of the entorhinal cortex. Thus, our data may also suggest the role of the entorhinal cortex in neuroticism-anxiety. The previous studies also showed a significant negative association between neuroticism and the FA value in the superior longitudinal fasciculus^8^, which is a pathway connecting the superior parietal and caudal middle frontal cortices^42^. Moreover, the middle fontal gyrus, including the caudal middle frontal cortex, has been proposed to be a site of convergence of the attention networks^43^. Neuroticism is associated with decreased attentional control over the visual field^44^.

Our results are also consistent with previous results using a graph theory analysis of rs-fMRI, which demonstrated that in highly neurotic individuals, the cingulo-operculum networks had relatively more connections with other functional networks, whereas the cognitive control networks in the default mode network showed less efficient information processing^14^. The cingulo-operculum networks consist of brain regions related to the identification and appraisal of salient affective stimuli and the production of affective states^45^. Likewise, previous studies have revealed that these high trait scorers dealt poorly with daily stressors and often applied maladaptive coping strategies, such as worrying and avoidance^46,47^. These findings implied that a neurotic brain is less cognitively controlled and that (negative) affect predominates in information processing. The cingulo-opercular network, which consists of the anterior insula/operculum, prefrontal cortex, dorsal anterior cingulate cortex, and thalamus^48^. Thus, the frontal pole and caudal anterior cingulate cortex, in which the neuroticism score was positively associated with the BET in our study, were included in the cingulo-opercular network. The default mode network consists of brain regions involved in attention, memory, emotion regulation, self-reflection, problem solving, and planning^49–51^. Hyatt *et al*. demonstrated that the default mode network was related to the mentalizing processes located in three core regions: the medial prefrontal cortex, the posterior cingulate cortex/precuneus, and the bilateral temporoparietal junction^52^. The isthmus cingulate and superior parietal cortices, which were detected in our study, are a portion of the posterior cingulate cortex and the precuneus, respectively.

In this study, in the left superior temporal cortex and left fusiform gyrus, the neuroticism score is also associated with the BET, which may consistent with the risk-conferring effects in social anxiety disorder (SAD). SAD, which is characterized by heightened fear of social evaluation in conjunction with a maladaptive pattern of emotion regulation^53,54^, has been associated with neuroticism^55^. SAD involves the impairment of networks associated with the ability to make inferences about others’ mental state, which has been termed ‘theory of mind’ (ToM)^56^. The superior temporal cortex is a brain structure that is thought to be important for social perception and mindreading based on the perception of emotions in facial stimuli^57,58^, and is also considered to play key roles in ToM processing. The fusiform gyrus also plays key roles in the pathology of SAD. SAD has been associated with hyper-reactivity in limbic brain regions like the amygdala, both during symptom provocation and emotional face processing tasks. Frick *et al*. showed that the severity in patients with SAD was positively correlated with amygdala connectivity with the fusiform gyrus^59^.

Our study is associated with some limitations. First, the small sample size must be taken into account when interpreting the results. Furthermore, the study participants were mostly adults with a mean age of 38 years and our findings cannot be applied to the general population. Second, we used DTI data with 25 non-collinear diffusion directions, which showed lower spatial resolution in comparison to previous studies^8,13^. Thus, our DTI sequence protocol might have limited the statistical power of the imaging analyses. Third, we could not verify an underlying altered functional network organization in the altered WM structural network that we observed because we only evaluated DTI data. Thus, we could not provide insight into the causal relationship between structural network organization and neuroticism. Future studies combining fMRI and DTI and/or following individuals longitudinally may provide additional insight. Furthermore, a more recent study proved the underlying genetic determinants associated with personality traits and mental health^60^. Thus, prospective studies combining genetic and structural brain metrics are also warranted to provide insight into the brain mechanisms underlying neuroticism in relation to psychiatric disorders.

In conclusion, we examined the alterations in the structural network organization or WM integrity associated with neuroticism. We found various brain regions that were both negatively and positively correlated with the neuroticism score. Our results, which are supported by a great deal of evidence from previous DTI and fMRI studies, suggest that various brain regions are related to neuroticism, and that the neuroticism trait is likely formed as a result of interactions among these regions.

### Compliance with Ethical Standards

Ethical approval: All procedures performed in studies involving human participants were in accordance with the ethical standards of the institutional and/or national research committee and with the 1964 Helsinki declaration and its later amendments or comparable ethical standards.

Informed consent: Informed consent was obtained from all individual participants included in the study.

## Data Availability

The datasets generated and analyzed during the current study are not publicly available due to restrictions set by the Ethics Committee of the University of Occupational and Environmental Health regarding patient confidentiality, but are available from the corresponding author on reasonable request.
